# Performance of Slow-Growing Male Muscovy Ducks Exposed to Different Dietary Levels of Quebracho Tannin

**DOI:** 10.3390/ani10060979

**Published:** 2020-06-04

**Authors:** Annelisse Castillo, Achille Schiavone, Maria Grazia Cappai, Joana Nery, Marta Gariglio, Stefano Sartore, Alessandro Franzoni, Margherita Marzoni

**Affiliations:** 1 Department of Veterinary Sciences, Università degli Studi di Torino, VIA Largo Paolo Braccini 2, 10095 Grugliasco (TO), Italy; annelisse.castillogarrido@unito.it (A.C.); achille.schiavone@unito.it (A.S.); marta.gariglio@unito.it (M.G.); stefano.sartore@unito.it (S.S.); alessandro.franzoni@unito.it (A.F.); 2 Department of Veterinary Medicine, Università di Sassari, VIA Vienna 2, 07100 Sassari (SS), Italy; mgcappai@uniss.it; 3 Department of Veterinary Sciences, Università di Pisa, VIA le delle Piagge 2, 56124 Pisa (PI), Italy; margherita.marzoni@unipi.it

**Keywords:** Muscovy duck, slow-growing, Quebracho tannin, growth performance, slaughter performance

## Abstract

**Simple Summary:**

Different inclusion levels of Quebracho tannin (QT) in the diet of growing male Muscovy ducks of a slow-growing type were explored under free-range conditions. As a result of the dietary treatments tested in this trial, the growth performance or the total blood proteins were not affected. By contrast, dietary QT did not lead birds to produce less moist excreta, as observed in other bird species, as a desirable aspect for intensively raised poultry. A marked improvement in carcass yield was observed as a desirable economic trait in the extensive slow-type duck farming system.

**Abstract:**

The study of the nutritional effects of tannins is complex due to the large chemical diversity; consequently, in poultry nutrition the biological responses may vary greatly. The aim of the present study was to evaluate the effect of different levels of dietary Quebracho tannins (QT) on growth and production performance in slow-growing type Muscovy ducks. For this purpose, a 42-d trial was carried out on 126 male ducks (42-d old at start), fed on three levels of dietary QT inclusion in the diet (0% as control diet, vs. 1.5% vs. 2.5% on an as fed basis). Birds were reared under free-range conditions. A linear increase in feed intake as a function of QT inclusion in the diet was observed (*p* < 0.05). No difference as to final body weight, overall average daily weight gain (ADG) and total feed conversion ratio (FCR) in relation to dietary treatments was observed. Carcass yields were positively improved in QT birds (*p* < 0.05). No adverse responses were recorded in total blood protein and liver weight. Dietary QT might be safely used up by to 2.5% in 42- to 84-d aged male Muscovy ducks.

## 1. Introduction

Tannins are plant secondary metabolites and represent the fourth most abundant group of secondary compounds of the plant kingdom, after cellulose, hemicellulose, and lignin [1]. Tannins are polyphenolic compounds, located mainly in vacuoles of the vegetal cell or in waxes, where they do not interfere with plant metabolism. They are found in many parts of the plant, such as fruits, leaves, bark, and wood [2], and in common foodstuffs, for example grapes, strawberries, blackberries, hazelnuts, cocoa, guarana. Feed such as sorghum grains, peas, fava beans, or legume trees like *Acacia* sp., *Sesbania* sp. also contain tannins [2,3], as well as acorns (*Quercus* spp.) and chestnuts (*Castanea sativa*), as spontaneous fruits of wood forest, chiefly for wild mammals and birds, but also domestic species [4,5,6]. Tannins exert various beneficial functions for the plant, depending on the tissue where they are found, such as regulating dormancy of seeds, contrasting pathogens acting as chemical barriers in roots and seeds, opposing predation of unripe fruits and leaves or preservation of the heartwood of conifers [2,3]. In some cases, tannins also attract insects towards flowers, thus helping in cross-pollination [7,8].

Tannins belong to a heterogenous group of phenolic compounds, with different chemical structures but with a high affinity to bind and precipitate proteins. Tannins are mainly classified into three major groups: hydrolysable tannins, condensed tannins or proanthocyanidins, and phlorotannins. The first two groups are found in terrestrial plants, whilst phlorotannins are found in marine brown algae [9,10]. Hydrolysable tannins are susceptible to hydrolysis by acids, bases or esterases, being easily degraded and absorbed in the digestive tract of mammals and birds [11,12]. Condensed tannins are oligomeric or polymeric flavonoids with complex structures and high molecular weights. In contrast to hydrolysable tannins, only strong oxidative and acidic hydrolysis can depolymerize the condensed tannin structures that are also not susceptible to anaerobic enzyme degradation [13]. Among the condensed tannin sources tested in poultry diets, we can find grape seed extract [14,15,16], grape pomace [16,17], mimosa [18] and Quebracho [19,20,21]. 

The phenolic structure of tannins is responsible for the antioxidant activity, which finds application in different fields, such as food industry and animal feeding, medical and pharmaceutical sectors [22]. In view of such technological and biological properties, tannins are considered an attractive family of chemicals, due to their various application potentials in different fields. Nevertheless, the study of the nutritional effects of tannins is complicated by the large chemical diversity [23], and consequently the bird’s responses may vary greatly. For instance, tannins were reported to act as anti-nutritional compounds in poultry diets, affecting productive performance due to a decrease in the feed intake and the digestibility of organic matter [24,25]. In contrast, it has been shown that depending on the bird’s age, health and physiological status, beneficial effects might be obtained by combining a specific tannin group in the diet. In this regard, a positive influence on chicken growth performance [26], improved final body weight and feed efficiency in broilers [27], reduced cholesterol level in Leghorn hens eggs [28], potential anticoccidial agent in broilers [19] and anthelmintic activity in pheasants [20] were reported in the reference literature.

Between the period 1998 and 2018, the global duck meat production expanded significantly, from 2.62 to 4.46 million tons, respectively [29]. Asia is the main global producer, accounting for 83.0% of the total duck meat output, followed by Europe, with 11.7% in 2018 [29]. The rearing of ducks follows different production systems. In developed countries for example, they are mainly reared intensively, while in Asia extensive production is largely applied [30]. Within duck species, the Muscovy duck (*Cairina moschata domestica,* Linnaeus, 1758.) represents an important economic resource. Central and South America represent the origin of this species, but currently Muscovy duck production is located mainly in Europe, with France as the first producer, together with southeast Asia and Taiwan [31]. Muscovy ducks are omnivorous birds, and the natural diet is based on worms, insects, fish, amphibians, reptiles, plants, and fruits [32]. For this reason, tannins, being widely distributed in the plant kingdom, might reasonably be part of their natural diet. Quebracho tannin (*Schinopsis* sp.) as a biologically active compound has been documented in pheasants and chickens [20,21], but limited information is available about the effects in the diet of Muscovy ducks. Therefore, the aim of the present study was to evaluate the effect of two inclusion levels of purified Quebracho tannin (QT) in the diet of slow-growing male Muscovy ducks on growth and production performance.

## 2. Materials and Methods

### 2.1. Birds and Diets

The experimental protocol (prot. no. 814715) was approved by the Bioethical Committee of the University of Turin (Italy). A feeding trial was carried out using one-hundred and twenty-six 42-d old males of Muscovy duck (*Cairina moschata domestica*, Linnaeus 1758), from a local slow-growing type, preserved since over 40 years at the Avian Conservation Center, which belongs to the Department of Veterinary Science of the University of Pisa (Italy). Birds were individually identified by a wing tag, weighted, and housed randomly in 18 roofed outdoor pens (6x3m) with sandy floor. Each pen housed seven animals.

Birds were allotted to the following experimental dietary groups: the control group (QT0) received the basal diet (Table 1); QT dietary groups received the same basal diet of QT0 with the inclusion of increasing QT levels at an amount of 1.5% and 2.5% on an as-fed basis, respectively. The QT is a commercial animal feed additive, extracted from the heartwood of *Schinopsis* sp., (MGM-S^®^, Unitan SAICA, Buenos Aires, Argentina). This product is available as a fine powder with 58% tannins, 20% phlobaphenes, 14% non-tannic compounds and 8% water and a polymerization degree of 6–7. Due to previous experiences in pheasants [20] and laying hens [21], the maximum safe level of QT to be used in diets for 42- to 84-d old Muscovy ducks is 2.5% on an as-fed basis, therefore, experimental groups were defined as group QT1.5 (2.6% MGM-S powder on top, corresponding to 1.5% QT) and QT2.5 (4.3% MGM-S powder on top, corresponding to 2.5% QT). For each period, the basal diet met or exceeded NRC [33] duck’s nutritional requirements and was formulated according to INRA [34] feed ingredient nutritional composition. Six replicates were assigned to each dietary treatment. Water and feed were supplied ad libitum. The chemical composition of the basal feed was analyzed in duplicate [35] and mean results are displayed in tables.

### 2.2. Growth Performance and Feed Conversion Efficiency

Growth performance was evaluated biweekly during the age interval 42–84 d. Individual live body weight (LBW) and feed intake per pen were recorded to calculate the average daily weight gain (ADWG), the average daily feed intake (ADFI) and the feed conversion ratio (FCR).

The LBW in each group were fitted by using LAB Fit-Curve Fitting Software [36]. To trace the first part of the growth curve, individual LBW, previously recorded, of all birds at 0, 14 and 28-d age was considered. The same data were then used in each of the three growth curves (QT0, QT1.5 and QT2.5). The Gompertz function resulted the best fitting growth curve. The following equation describes the curve

$$W_{\left(t\right)}\;=\;W_{¥}\;e^{{-e}^{-k\;(t\;-\;t{}^{\circ})}}$$

where W_(t)_ is the weight in grams of duck at time t, W_¥_ is the asymptotic final LBW, k is the constant expressing the rate of approach to W_¥_, and t° is the age at the inflexion point where maximum rate of growth is achieved. The maximum rate of growth for each group was calculated by the differential equation of 1° grade derived from Gompertz

$$W^{\prime }\left(t\right)\;=\;k\cdot W_{¥}\;e^{{-e}^{-k\cdot (t\;-\;t{}^{\circ})}-k\cdot (t\;-\;t{}^{\circ})}$$

The values of W_¥_, k and t° are the same described above for the growth curve, W’(t) is the daily weight increase in grams at time t [37].

### 2.3. Blood Sampling

Blood samples from 24 birds (84 d old) from each dietary group (four birds/pen) were collected during slaughter procedures into K_3_EDTA 2mL vacuum tubes. Total blood protein (TBP) was determined according to Salamano et al. [38].

### 2.4. Excreta Sampling and Dry Matter

Excreta sample collection was carried out on 24 ducks (81 d old) per dietary treatment, following the procedure described by Gariglio et al. [39]. Briefly, the birds were kept and temporary housed (1 h) in cages (two birds in the same pen/cage). Excreta samples free from debris and foreign substances were collected from a tray placed under each cage. A total of 12 pooled samples per dietary treatment were collected at each sampling. The dry matter (DM) of excreta was determined according to AOAC [35].

### 2.5. Slaughter Traits and Organs Evaluation

Twelve birds (84 d old) per dietary treatment were randomly chosen and fasted for 12-h, to be finally weighed and slaughtered in a commercial abattoir. Birds were electrically stunned prior to bleeding. All bird carcasses were mechanically plucked after immersion in hot water. Evisceration was performed manually. Fresh absolute weight of liver and gizzard were determined, and relative weight expressed as percentage of live body weight (LBW). Head-neck and feet were removed and weighed separately; the respective relative weights were calculated as percentage of LBW. Hot weight of ready-to-cook carcass (RCC) was recorded and expressed as percentage of LBW. The small intestine and caeca were unfolded on a flat surface to obtain a fully extended viscera and allow linear length measurement; the respective lengths were expressed as cm per 100g of LBW. Carcasses were stored in a cool chamber at 4 ± 0.5 °C for 24 h, then chilled RCC weights were determined and expressed as percentage of LBW. Finally, *m. pectoralis* and legs + thigs were separated from the RCC and weights were determined and expressed as percentage of LBW.

### 2.6. Statistical Analysis

The statistical analyses were performed using the SPSS software [40]. Shapiro–Wilk’s test established normality or non-normality of distribution, and the assumption of equal variances was assessed by means of Levene’s homogeneity of variance test. The experimental unit was the pen. The collected data were tested by means of one-way ANOVA, using polynomial contrasts to test the linear and quadratic responses to increases in the QT inclusion levels in the diet. For all tests, *p* < 0.05 was considered statistically significant. Results were expressed as the mean and standard error of the means (SEM). A statistical trend was considered for *p* < 0.10.

## 3. Results

### 3.1. Growth Performance and Feed Conversion Ratio

Throughout the experimental period, no mortality was experienced. Bird performance is summarized in Table 2. Overall LBW was not influenced by dietary treatments (*p* > 0.05), except for 70-d age, where a quadratic response was observed, and the minimum shown by QT1.5 group (*p* < 0.05).

Dietary QT negatively influenced ADWG during the middle part of the experimental period (56–70 d), showing a quadratic response (*p* < 0.01). The following period (70–84 d) instead, was positively influenced by QT, showing linear and quadratic responses with a minimum for QT0 group (*p* < 0.01).

The ADFI resulted directly proportional to the concentration of QT during the 56–70 period, showing a linear (*p* < 0.01) and a quadratic (*p* < 0.05) response. A linear increase (*p* < 0.05) was also observed when considering the whole experimental period (42–84 d). The FCR was not affected by dietary QT, considering both the single age periods, and the whole experimental period.

According to the calculated Gompertz parameters (Table 3), birds reached the inflection point when this trial began (42-d age). Curve parameters were close to each other (Table 3).

### 3.2. Total Blood Proteins

TBP in 84-d old birds are reported in Table 4. The dietary QT inclusion did not influence the level of TBP (*p* > 0.05). In birds, normal TBP concentrations ranged between 3.5–5.5 g/dl [41]. Results in this study placed all birds within this range.

### 3.3. Excreta Dry Matter Evaluation

Excreta dry matter content in 81-d old birds is reported in Table 5. The dietary QT inclusion did not influence this parameter (*p* > 0.05). 

### 3.4. Slaughter Traits

Slaughter traits in 84-d old male Muscovy ducks are reported in Table 6. The dietary QT inclusion had no influence on the development of breast muscles and Legs + thigs (*p* > 0.05). Both RCC, hot and chilled, were positively influenced by dietary treatment, showing a linear (*p* < 0.05) and a quadratic response (*p* < 0.01). Dietary treatment affected linearly the Head-Neck mass, which decrease by the increase level of dietary QT (*p* < 0.05). All other slaughter traits were not affected by the dietary treatment (*p* < 0.05), except for the SIL, which evidenced a quadratic response, with the minimum value expressed by the lowest QT inclusion level (*p* < 0.01).

## 4. Discussion

The increasing interest in the use of some plant-derived molecules with biological properties offers the chance to exploit several beneficial effects due to their numerous properties [9,42]. Against this background, more information seems to be needed about their safe use in different species, to achieve the desired effects. From this perspective, and due to previous experiences in pheasant [20] and pullets [21], the maximum safe QT level to be used in diets for 42 to 84-d old Muscovy ducks was set to 2.5%, on an as-fed basis. At this QT amount, no adverse effects were observed in birds of this trial, as supported by the TBP levels and liver weight. On the contrary, in a previous study involving slow-growing pullets [21], adverse effects were observed in 35-d old birds fed 3% dietary tannins supplementation, resulting in 2% of QT as the safe inclusion level. It is widely known that tannins bind and precipitate proteins, which compromises absorption at intestinal level. This situation might lead to protein deficiency in the bloodstream [9]. Ducks in this study fed on QT diets did not exhibit a decrease in TBP circulating value. Additionally, our results agree with those obtained by Gariglio et al. [43] in 50-d old female Muscovy ducks.

As to liver, weight variations can be useful signs of the presence of hepatic injuries [44]. In this trial, liver weights in QT birds were similar across birds from all experimental groups, at each QT inclusion rate tested. Furthermore, at the end of the experimental period, birds of QT1.5 and QT2.5 groups reached a similar LBW at slaughter. As demonstrated also by the growth curve trends, which were almost identical, it can be stated that the tested amounts of QT included in the diet did not negatively impact the production performance of slow-growing male Muscovy ducks. In agreement with other authors, who reported duck’s growth curves using Gompertz model [45,46], we also found this model as the best fitting in our trials (R^2^ = 0.98–0.99).

According to the QT inclusion levels tested, a different reaction in birds was noted regarding feed intake. Birds of the QT1.5 group needed a longer time to get used to the change in diet, as demonstrated during the first days after dietary QT inclusion with an evident decrease in the ADWG and ADFI. Birds of the QT2.5 group, instead, seemed to cope with the QT by increasing the ADFI, which was higher during the whole experimental period. Such differences in response to different levels of dietary QT inclusion might be explained by the natural feeding behavior of the Muscovy duck, for which a large part of foraging activity takes place in fresh water [32]. In captivity, when ducks find a hard, unusual or an unknown tasty food, they use to soak it into water in order to render it “eatable”. It could be argued that such behavior of soaking feeds was performed with the purpose of expressing the innate feeding behavior. With the increasing QT, the feed taste becomes reasonably astringent and birds tended to put the feed into the water with a consistent higher consumption. In QT1.5 group, instead, astringency perception presumably following a lower QT inclusion amount, might have not led birds to adopt coping solutions, for which a lower feed intake was also observed. Elkin et al. [47] reported no growth depression in ducks fed sorghum rich in tannins. Same authors also suggested that the innate feeding behavior of ducks could be a key factor in the interpretation of such a response. 

In this trial, the FCR calculated throughout the experimental period and the final LBW were not affected by dietary QT at both tested levels. However, in QT1.5 birds, an initial retarded growth occurred, with a successive compensatory growth, evident especially during the last 15 days (70–84). This compensatory growth trend was also reported by other authors in broilers reared under feeding restriction, followed by ad libitum feed provision [48]. A decrease in growth rate, lower LBW, lower ADFI and higher FCR were reported in male Muscovy ducks fed low- or high-tannin-sorghum diets [49]. These authors also reported no differences in these parameters in Muscovy males fed on diets 50/50 high/low tannin-sorghum enriched with L-Methionine, attributing the reduction in the negative effect of tannin on protein availability to this amino acid.

Unexpectedly, the use of dietary QT did not lead birds to excrete dryer droppings as observed in other bird species, namely chickens [21] and pheasants [20], but rather the opposite effect could be observed. This result might be attributed to the high daily water intake in ducks, which did not allow the QT to have a “drying” effect. As some authors reported, 800 ml/d was the water intake in 14 to 42-d old Pekin ducks [50], while in 20-week old White Leghorn hens the intake was 228 ml/d [51], thus, threefold lower than the intake in ducks.

The dietary QT did not negatively affect carcass traits in 84-d old male ducks, rather, it led to improved yields in both the hot and chilled RCC of QT birds, which was heavier than QT0. Higher RCC yields were also reported in male Muscovy ducks of the same strain [52], fed on diets with 50/50 high/low tannin-sorghum [48]. In slow-growing chickens, instead, the RCC yield pointed out no differences [21], as well as in broiler chickens [26] and in female pheasants [20]. 

Wild birds feeding on diets rich in tannins developed larger intestines and caeca, and heavier gizzards [53], however, this could be also induced by a different physical form of natural feeding sources, rich also in more fibrous nutrient content [54]. In this trial, small intestine length was influenced by the diet. Nevertheless, this influence resulted in the opposite to what we would have expected, with QT1.5 birds having a shorter small intestine. In contrast, no effect was observed in growing female pheasants after a 60-d QT diet [20], and longer small intestine length after a 4-week trial in adult grey partridges was observed by Liukkonen-Anttila et al. [53]. Further research is needed to clarify this result.

## 5. Conclusions

In view of the parameters tested in this feeding trial, no adverse effects on health nor on the production performance of birds could be observed. The increasing amount of QT in the diet was found to be linked to the higher intake of feed by the ducks of the different dietary groups, likely due to the capability of dietary quebracho tannins to stimulate the innate feeding behavior. Excreta quality was not influenced by the amount of tannins in the diet, probably due to a conserved water consumption via soaked feed. Finally, the slaughtering performance was satisfactory and improved by QT supplementation, thus the inclusion up to 2.5% of Quebracho tannins in the diet can be safely used in slow-growing type male Muscovy ducks.

## Figures and Tables

**Table 1:** Ingredients and chemical composition of basal diet.

Ingredient (%)	Days	
	0–41	42–84
Corn meal	64.10	67.30
Soybean meal	16.00	10.00
Bran	3.63	6.62
Corn gluten meal	9.00	9.00
Soybean oil	2.85	3.45
Dicalcium phosphate	1.30	0.40
Calcium carbonate	1.40	1.74
Sodium chloride	0.25	0.25
Sodium bicarbonate	0.20	0.20
DL-Met	0.17	0.03
L-Lys	0.39	0.30
Vitamin-mineral premix ^1^	0.50	0.50
Choline cloride	0.01	0.01
Optifos 250 bro ^2^	0.10	0.10
Avizyme 1500 x ^3^	0.10	0.10
Chemical composition		
Dry matter (DM), %	88.80	88.70
Crude protein, % DM	23.00	20.20
Ether extract, % DM	6.20	7.30
Neutral detergent fiber, % DM	12.90	12.70
Ash, % DM	7.80	6.50
Metabolizable energy, MJ/kg ^4^	12.53	12.77

**^1^** Supplied per kg of diet: Vit. A 62.5 IU; Vit. D3 17.5 IU; Vit. E 200 µg; Vit. K 10 µg; Biotin 1 µg; Thiamine 10 µg; Riboflavin 30 µg; Pantothenate 76.05 µg; Niacin 200 µg; Choline 3750 µg; Pyridoxine 20 µg; Folic acid 3.75 µg; Vit. B12 0.15 µg; Mn 350 µg; Zn 310.75 µg; Fe 250 µg; Cu 350 µg; I 1.25 µg; Se 1.25 µg; **^2^** Phytase (EC3.1.3.26) (250 OTU/kg diet), Huvepharma, Sofia, Bulgaria; **^3^** Complex of Endo 1-4-β- Xylanase (EC3.2.1.8) (256 U/kg), Subtilisin (EC3.4.21.62) (2560 U/kg diet) and α-Amylase (EC3.2.1.1) (1472 U/kg diet), Danisco Animal Nutrition, Marlborough, Wiltshire, UK; **^4^** Based on INRA [34] ingredient composition.

**Table 2:** Growth performance of Muscovy ducks fed different levels of dietary Quebracho tannin (QT) from 42 to 84-d age (*n* = 6 pens /treatment; 7 birds/pen).

Items	Age	Diet ^1^			SEM	*p*-Value	
		QT0	QT1.5	QT2.5		Linear	Quadratic
LBW, g	42 d	1699	1653	1676	14.6	0.541	0.259
	56 d	2703	2667	2635	16.4	0.094	0.949
	70 d	3408	3272	3330	20.6	0.126	0.023
	84 d	3787	3784	3789	19.1	0.956	0.913
ADWG, g/d	42–56 d	71.8	72.4	68.5	0.77	0.084	0.147
	56–70 d	50.3	43.2	49.7	0.69	0.688	0.000
	70–84 d	27.0	36.5	32.8	0.75	0.001	0.000
	42–84 d	49.7	50.73	50.3	0.42	0.571	0.414
ADFI, g/d	42–56 d	241	242	259	4.09	0.063	0.266
	56–70 d	232	227	269	7.44	0.010	0.036
	70–84 d	221	228	239	5.23	0.188	0.834
	42–84 d	231	232	256	4.75	0.014	0.116
FCR, g/g	42–56 d	3.36	3.34	3.83	0.11	0.077	0.230
	56–70 d	4.61	5.28	5.46	0.20	0.090	0.536
	70–84 d	8.72	6.31	7.35	0.45	0.147	0.052
	42–84 d	4.68	4.58	5.11	0.11	0.114	0.172

^1^ QT0 = basal diet; QT1.5 and QT2.5 = the basal diet supplemented with QT at 1.5% and 2.5%, respectively. Note: SEM: standard error of the mean; LW: live body weight; ADWG: average daily weight gain; ADFI: average daily feed intake; FCR: feed conversion ratio.

**Table 3:** Estimated Gompertz growth parameters, inflection point, and goodness of fit in male Muscovy ducks fed different levels of dietary Quebracho tannin (QT).

Diet ^1^	W_¥_ (g) ^2^	K ^3^	t° (d) ^4^	Maximum ADWG (g/d)	Weight at Inflection Point (g)	R^2^
QT0	4470	0.0444	42	73	1677	0.98
QT1.5	4425	0.0436	42	71	1662	0.99
QT2.5	4475	0.0431	42	71	1657	0.98

^1^ QT0 = basal diet; QT1.5 and QT2.5 = the basal diet supplemented with QT at 1.5% and 2.5%, respectively; ^2^ W_¥_ = asymptotic final LBW; ^3^ k = constant, expressing rate of approach to W_¥_; ^4^ t° = age at inflexion point; ADWG: average daily weight gain.

**Table 4:** Total blood protein (TBP) of Muscovy ducks fed different levels of dietary Quebracho tannin (QT) at 84-d age (*n* = 6 pen/treatment; 4 birds/pen).

Items	Age	Diet ^1^			SEM	*p*-Value	
		QT0	QT1.5	QT2.5		Linear	Quadratic
TBP %	84 d	4.50	4.33	4.38	0.05	0.351	0.319

^1^ QT0 = basal diet; QT1.5 and QT2.5 = the basal diet supplemented with QT at 1.5% and 2.5%, respectively.

**Table 5:** Excreta dry matter (DM) of Muscovy ducks fed different levels of dietary Quebracho tannin (QT) at 84-d age (*n* = 6 pen/treatment; 4 birds/pen).

Items	Age	Diet ^1^			SEM	*p*-Value	
		QT0	QT1.5	QT2.5		Linear	Quadratic
DM %	84 d	45.3	47.4	49.0	1.89	0.448	0.949

^1^ QT0 = basal diet; QT1.5 and QT2.5 = the basal diet supplemented with QT at 1.5% and 2.5%, respectively.

**Table 6:** Slaughter traits of male Muscovy ducks fed different levels of Quebracho tannin (QT) from 42-d to 84-d age (*n* = 6 pens /treatment; 2 birds/pen).

Items		Diet ^1^			SEM	*p*-Value	
		QT0	QT1.5	QT2.5		Linear	Quadratic
LBW	g	3782	3793	3809	26.0	0.678	0.963
Breast	% of LBW	12.48	13.16	12.55	0.21	0.897	0.160
Legs + thighs		12.89	12.90	12.79	0.10	0.687	0.776
RCC_hot_		59.6	62.6	61.0	0.33	0.019	0.000
RCC_chilled_		58.5	61.5	60.1	0.32	0.014	0.000
Head-Neck		10.3	10.1	9.5	0.16	0.035	0.531
Feet		2.74	2.66	2.67	0.03	0.247	0.447
Liver		1.80	1.64	1.74	0.04	0.589	0.143
Heart		0.73	0.67	0.70	0.01	0.405	0.236
Gizzard		1.91	1.83	1.87	0.03	0.620	0.400
Intestine		4.87	4.71	4.76	0.13	0.740	0.691
SIL	cm/100 g LBW	5.52	5.11	5.33	0.06	0.148	0.013
CL		1.14	1.05	1.06	0.02	0.053	0.171

^1^ QT0 = basal diet; QT1.5 and QT2.5 = the basal diet supplemented with QT at 1.5% and 2.5%, respectively. Note: SEM: standard error of the mean; LBW: live body weight; RCC: ready to cook carcass; SIL: small intestine length; CL: caeca length.
